# Supplementary material for: WordCommentsAnalyzer: A windows software tool for qualitative research
Source: F1000Res. 2018 Sep 4;7:536. Originally published 2018 May 3. [Version 2] doi: 10.12688/f1000research.14819.2 (PMC6137411; doi:10.12688/f1000research.14819.2)
Supplement: Supplementary file 3 [file f1000research-7-17614-s0002.tgz › b2a79496-4ada-47c4-9682-a99ce867836d.zip!/Eight Iranian Health Journals/Journal of Research in Rehabilitation Sciences _40.docx]

© Journal of Research in Rehabilitation Sciences . This work is licensed under CC BY-NC 4.0 (https://creativecommons.org/licenses/by-nc/4.0/).

Volume & Issue: دوره 8، شماره 1: 1391

Title: بررسی حس عمقی مفصل زانو در تحرک بیش از حد عمومی مفصلی و مقایسه آن با افراد سالم

http://jrrs.mui.ac.ir/index.php/jrrs/article/view/170

مقدمه: نقش تحرک بیش از حد مفصلی (هایپرموبیلیتی مفصلی) در بروز آسیب‌های عضلانی- اسکلتی مورد تأکید قرار گرفته است. در مطالعات اخیر نقایص حس عمقی در افراد مبتلا به این سندرم گزارش گردیده است. از آن جا که افراد مبتلا به سندرم فوق بر خلاف تحرک بیش از حد عمومی دارای ضایعات عضلانی- اسکلتی می‌باشند که خود ممکن است منشأ اختلال حس عمقی باشد، وجود این گزارش‌ها نمی‌تواند به طور خالص تأثیر هایپرموبیلیتی را بر حس عمقی تأیید نماید. بنابراین هدف از انجام این مطالعه، بررسی تأثیر تحرک بیش از حد عمومی بر حس وضعیت مفصل زانو در این افراد بود. مواد و روش‌ها: در این مطالعه موردی- شاهدی، 20 فرد دارای تحرک بیش از حد عمومی که دارای تحرک بیش از حد زانوی غالب بودند و 20 فرد سالم به روش نمونه‌گیری ساده در دسترس از بین دانشجویان دختر و پسر در محدوده سنی 18 تا 30 سال انتخاب شدند. خطای حس وضعیت در سه زاویه 15-، 40- و 65- درجه اکستنشن زانو به روش اکتیو در حالت نشسته به کمک گونیامتری تصاویر دیجیتال توسط نرم‌افزار اتوکد اندازه‌گیری شد و بین افراد دو گروه و همین طور بین زوایای مختلف مقایسه گردید. یافته‌ها: دقت حس وضعیت مفصل زانوی جوانان مبتلا به تحرک بیش از حد عمومی مفصلی کمتر از افراد سالم بود. همچنین حس وضعیت مفصل زانوی هر دو گروه در زوایای انتهایی از دقت بیش‌تری نسبت به زوایای داخلی‌تر برخوردار بود. نتیجه گیری: مطالعه حاضر وجود نقص حس وضعیت مفصل زانو را در افراد دچار تحرک بیش از حد عمومی به اثبات می‌رساند. بنابراین ارزیابی حس عمقی مفاصل در این افراد، به خصوص زمانی که قصد دارند وارد آن دسته از فعالیت‌های ورزشی گردند که به دنبال آن خطر صدمات مفصلی افزایش می‌یابد، لازم خواهد بود. همچنین طراحی مطالعات مداخله‌ای به منظور بررسی پروتکل‌های تمرینی برای بهبود حس وضعیت مفاصل این افراد ضروری است.

Title: مقايسه استفاده از زمان و رضایت از مدیریت زمان در مادران کودکان فلج مغزی با مادران كودكان سالم

http://jrrs.mui.ac.ir/index.php/jrrs/article/view/185

مقدمه: مطالعه الگوهای استفاده از زمان نقش مثبتی در سلامت و رفاه افراد دارد. به علاوه آگاهی ما را در رابطه با میزان مشارکت در کارها افزایش می‌دهد. داشتن یک کودک فلج مغزی همواره فشارهایی را بر خانواده وارد می‌سازد، که یکی از آن‌ها نیاز به مراقبت دایمی است که زمان مورد نیاز آن فراتر از برنامه زمان‌بندی خانواده است. این حیطه که در اکثر موارد مادران را به عنوان مراقبین اصلی درگیر می‌کند، کمتر مورد توجه قرار گرفته است. لذا جهت گسترش آگاهی در این زمینه و همچنین برنامه‌ریزی‌های آینده، در این پژوهش الگوهای استفاده از زمان در مادران کودکان فلج مغزی مورد بررسی قرار گرفته است. مواد و روش‌ها: برای رسیدن به هدف اصلی، مطالعه حاضر از نوع مورد- شاهد انتخاب شد. نمونه‌گیری به روش نمونه‌گیری در دسترس بود. در گروه مادران دارای کودکان فلج مغزی 30 نفر و در گروه مادران کودکان سالم 37 نفر شرکت کننده وجود داشت. داده‌های لازم برای پژوهش با استفاده از پرسش‌نامه "استفاده از زمان مادران" و نیز پرسش‌نامه دموگرافیک جمع‌آوری گردید. از آزمون‌های Independent t-test و Mann–Whitney برای تحلیل داده‌ها در 14SPSS استفاده گردید. یافته‌ها: آنالیز آماری تفاوت معنی‌داری را در حیطه کاری مراقبت از کودک نشان داد (05/0 > P)، اما در سایر حیطه‌های کاری تفاوت معنی‌داری به دست نیامد (05/0 < P). در میزان رضایت از مهارت مدیریت زمان نیز تفاوت معنی‌داری به دست آمد؛ به طوری که مادران کودکان سالم رضایت بیش‌تری داشتند (05/0 > P). نتیجه گیری: یافته‌های حاصل از این مطالعه نشان داد که مادران کودکان فلج مغزی به دلیل آن که زمان بسیار زیادی را در روز صرف مراقبت از فرزندان خود می‌کنند، توازن لازم را در حیطه‌های کاری مختلف خود تجربه نمی‌کنند. این امر منجر به رضایت کمتر از "استفاده از زمان" می‌گردد. با توجه به این یافته‌ها لازم است که منابع حمایتی و مراقبتی بیش‌تری در اختیار این افراد قرار داده شود، تا با گذر زمان احتمال مواجه شدن با مسایل روانی و مشکلات مربوط به سلامتی کمتری وجود داشته باشد.

Title: بررسي و مقايسه تکرارپذيری پايداری وضعيتي حين حفظ بار ديناميکي در مردان سالم و مبتلا به کمردرد مزمن غير اختصاصی

http://jrrs.mui.ac.ir/index.php/jrrs/article/view/301

مقدمه: کمردرد ناشی از فعاليت‌‌های باربرداری و نگهداشتن بار عامل مهمی در ايجاد ناتوانی عملکردی برای بيمار و ضررهای سنگين اقتصادی است. هنگام نگهداری بارهای متغير ديناميکي مقدار گشتاور وارده به ستون فقرات در حال تغيير است که می‌تواند تعادل را به مخاطره بيندازد. هدف از اين تحقيق، بررسی تکرارپذيری پايداری وضعيتي حين حفظ بار متغير ديناميکی به منظور ارزيابی دقيق‌تر ضايعات ستون فقرات کمری بود. مواد و روش‌ها: 24 مرد شامل 12 فرد مبتلا به کمردرد و 12 فرد سالم در اين مطالعه شرکت کردند. افراد بارهای ديناميکی را در دو وضعيت ايستاده و خميده تنه (8 حالت)، در زمان‌های مختلف يک جلسه آزمايش حفظ کردند. تکرارپذيری (Reliability) داده‌های صفحه نيرو شامل دامنه جابجايی (Displacement) و سرعت (Velocity) مرکز فشار (Center of pressure یا COP) و انحراف معيار آن‌ها در دو جهت قدامی- خلفی و طرفی طول مسير مرکز فشار مورد ارزيابی قرار گرفت. تکرارپذيری نسبی با استفاده از ضريب همبستگی درون گروهی (Intraclass correlation coefficient یا ICC) و تکرارپذيری مطلق با استفاده از خطای معيار اندازه‌گيری (SEM  یا Standard error of measurement) و حداقل تغيير قابل اندازه‌گيري (Minimal metrically detectable change یا MMDC) محاسبه گرديد. یافته‌ها: از ميان پارامترهای مرکز فشار، سرعت مرکز فشار در جهت طرفی در افراد سالم (9/0-8/0)، سرعت و انحراف معيار سرعت مرکز فشار در جهت قدامی- خلفی در افراد مبتلا به کمردرد (9/0-7/0)، تکرارپذيری بالايی را نشان دادند. تکرارپذيری دامنه جابجايی مرکز فشار در جهت قدامی– خلفی و انحراف معيار سرعت مرکز فشار در جهت طرفی در هر دو گروه مشابه بود (9/0-6/0). تکرارپذيری دامنه جابجايی مرکز فشار و انحراف معيار آن در جهت طرفی و نيز طول مسير مرکز فشار در افراد سالم بيشتر از افراد مبتلا به کمردرد بود، ولي تفاوت معني‌دار نبود. نتیجه گیری: به نظر می‌رسد سرعت مرکز فشار در دو جهت قدامی- خلفی و طرفی پارامتر مناسبی جهت ارزيابی پايداری وضعيتي حين حفظ بار ديناميکی در افراد مبتلا به کمردرد و افراد سالم باشد.

Title: مقایسه کارکردهای اجرایی کودکان اتیستیک با عملکرد بالا و همتایان عادی

http://jrrs.mui.ac.ir/index.php/jrrs/article/view/307

مقدمه: فرضیه‌های متعددی در خصوص نقصان کارکردهای شناختی در کودکان مبتلا به اختلال طیف اتیسم وجود دارد که یکی از آن‌ها فرضیه نقصان کارکردهای اجرایی است. هدف از این مطالعه، بررسی تفاوت دو گروه کودکان مبتلا به اتیسم و همتایان سالم در توجه پایدار، توجه انتخابی و مهار پاسخ به عنوان کارکردهای اجرایی بود. مواد و روش‌ها: در این مطالعه مقطعي، 16 کودک مبتلا به اتیسم با عملکرد بالا با 16 کودک همتای عادی مورد مقايسه قرار گرفتند. آن‌ها به انجام آزمون استروپ رنگ- واژه (جهت ارزیابی توجه انتخابی) و آزمون برو نرو (جهت ارزیابی مهار) و آزمون عملکرد مداوم (جهت ارزیابی توجه پایدار) پرداختند. جهت آنالیز داده‌ها Independent t-test مورد استفاده قرار گرفت. از نرم‌افزار 18SPSS برای آناليز داده‌ها استفاده شد و سطح معنی‌داری 01/0 در نظر گرفته شد. یافته‌ها: در آزمون استروپ در تعداد پاسخ‌های صحیح (05/0 < P) و میانگین زمان (05/0 < P) در دو گروه اتیسم و عادی تفاوت معنی‌داری وجود نداشت. در آزمون عملکرد مداوم در خطای حذف (05/0 < P) و خطای تکانش‌گری (05/0 < P) بین دو گروه اتیسم و عادی تفاوت معنی‌داری مشاهده نشد. در حالی که در همین تکلیف زمان پاسخ در گروه اتیسم به طور معنی‌داری (01/0 > P) کمتر از همتایان عادی بود. در آزمون برو نرو، میانگین زمان (05/0 < P) در مرحله برو، میزان پاسخ‌های صحیح (05/0 < P) و میانگین زمان (05/0 < P) در مرحله نرو در دو گروه اتیسم و عادی تفاوت معنی‌داری نداشت. در حالی که میزان پاسخ‌های صحیح در مرحله برو (01/0 > P) در گروه عادی به طور معنی‌داری بیشتر از این میزان در گروه اتیسم بود. نتیجه گیری: مهار پاسخ (غالب و حرکتی) و توجه پایدار از جمله کارکردهای اجرایی شناختی سالم در افراد اتیسم با عملکرد بالا است. سلامت مهار پاسخ در اتیسم می‌تواند به علت نقص این افراد در درک مطلب، مناطق مغزی درگیر و یا نوع تکالیف ارایه شده باشد. سلامت توجه پایدار در افراد اتیسم نیز حاکی از تمایل به تمرکز روی محرک‌های خاص و رفتارهای تکراری است که از علایم شناخته شده اتیسم می‌باشد.

Title: اثر تمرينات ثبات ‌دهنده مركزي بر درد و عملكرد بیماران زن مبتلا به استئوآرتريت زانو

http://jrrs.mui.ac.ir/index.php/jrrs/article/view/282

مقدمه: زانو به دليل تحمل وزن بدن در معرض مستقيم ضربه قرار دارد و به اين دليل بيش‌ترين شيوع استئوآرتريت را به خود اختصاص داده است و علایمي مانند درد، خشكي و كاهش شديد حركات مفصلي را به همراه دارد. هدف از پژوهش حاضر، ارزیابی تأثير يک دوره تمرين ثبات‌ دهنده مركزي بر درد و عملكرد بیماران زن مبتلا به استئوآرتريت زانو بود. مواد و روش‌ها: تعداد 22 بیمار زن غير ورزشكار مبتلا به استئوآرتريت زانو (ميانگين سني 38/9 ± 12/60 سال، قد 84/7 ± 45/155 سانتی‌متر و وزن87/13 ± 46/71 كيلوگرم) انتخاب و به صورت تصادفي در دو گروه تجربي و شاهد تقسيم شدند. در ابتدا ميزان درد بيماران از طريق پرسش‌نامه VAS (Visual analogue scale) و عملكرد آن‌ها از طریق پرسش‌نامه WOMAC (Western ontario and mcmaster universities index) اندازه‌گيري شد. سپس گروه تجربي برای مدت 8 هفته، هفته‌اي 3 جلسه، هر جلسه 30 دقيقه تحت تمرينات ثبات ‌دهنده مركزي قرار گرفتند. بیماران گروه شاهد به روال عادی زندگي خود ادامه دادند. براي تجزيه و تحليل اطلاعات از ANOVA One way استفاده شد. یافته‌ها: نتايج بهبود معنی‌داري در ميانگين درد قبل (27/2 ± 81/4) و بعد (72/1 ± 18/3) از تمرين بیماران گروه تجربي نشان داد (05/0 > P). اما تفاوت بين ميانگين درد قبل (21/2 ± 09/5) و بعد (04/2 ± 81/6) از تمرين در گروه شاهد معنی‌دار نبود (05/0 < P). همچنين در ميانگين عملكرد قبل (89/17 ± 72/41) و بعد (35/17 ± 72/28) از تمرين بیماران گروه تجربي بهبود معنی‌داري مشاهده شد (05/0 > P). ولی بهبود ميانگين عملكرد قبل (82/14 ± 45/44) و بعد (03/12 ± 09/52) از تمرين گروه شاهد معنی‌دار نبود (05/0P > ). نتیجه گیری: از نتايج تحقيق می‌توان نتیجه‌گیری کرد که تمرينات ثبات ‌دهنده مركزي در بهبود درد و عملكرد مفصل زانو تأثير شایانی داشته است. از آن جايي كه عملكرد مناسب عضلات مركزي نقش اساسي در قرار گرفتن لگن در وضعيت خنثي دارد، اين حالت ممكن است بتواند منجر به قرارگيري استخوان ران در وضعيت طبيعي و در نتيجه وضعيت خوب مفصل زانو و كاهش فشارهاي وارده بر غضروف مفصلي گردد. لذا پيشنهاد می‌شود که از تمرينات ثبات‌ دهنده مرکزی به عنوان يک روش مؤثر در پيش‌گيري و درمان استئوآرتريت زانو استفاده گردد.

Title: روایی و پایایی نسخه فارسی آزمون "ارزيابي جامع تجربه فرد از لكنت- بزرگ‌سالان"

http://jrrs.mui.ac.ir/index.php/jrrs/article/view/433

مقدمه: به منظور به كارگيري يك رويكرد به طور کامل مبتني بر شواهد در اختلال لکنت، نياز به ابزاري است كه كليت اين اختلال را مورد بررسي قرار دهد. براي دست يافتن به اين مهم، Yaruss و Quesal در سال 2006 ابزاري را تحت عنوان "ارزيابي جامع تجربه فرد از لكنت" ارایه كردند. این پرسش‌نامه مي‌تواند به عنوان ابزاري براي مستند كردن نتايج چندگانه در درمان لكنت افراد بالاي 18 سال مورد استفاده قرار گيرد. هدف اين تحقيق، تعيين روايي و پایایی نسخه فارسي شده پرسش‌نامه فوق بود. مواد و روش‌ها: این مطالعه بر روی 60 فرد بزرگ‌سال دارای لکنت به اجرا در آمد. با اجرای یک مطالعه آزمایشی بر روی 10 فرد دارای لکنت و انجام یک مصاحبه نیمه ساختمند روايي صوري، ثبات درونی و اعتبار پرسش‌نامه ارزيابي جامع تجربه فرد از لكنت بررسی شد. بعد از این مرحله 50 فرد دارای لکنت پرسش‌نامه تعدیل یافته را به همراه آزمون نگرش ارتباطی، به منظور بررسی روایی همزمان، تکمیل نمودند. در پایان نسخه فارسی پرسش‌نامه، توسط 5 فرد دارای لکنت در دو بار به فاصله 7 تا 10 روز به منظور بررسی اعتبار آزمون- باز آزمون تکمیل گردید. یافته‌ها: ضرايب Cronbach's alpha، كه به طور مستقل براي هر يك از چهار بخش اين ابزار محاسبه شد، پايایي دروني از 82 تا 97 درصد را نشان داد. بنابراین نسخه فارسی پرسش‌نامه "ارزيابي جامع تجربه فرد از لكنت" از روایی همزمان، هم‌خواني دروني و تكرارپذيري نسبي و در كل از پايايي مطلوبي نیز برخوردار بود. نتیجه گیری: می‌توان اظهار داشت که نسخه فارسي توانسته است همگني در گزینه‌های نسخه اصلي را حفظ نماید و از اين جنبه مي‌توان اميدوار بود كه ابزار به ‌دست آمده با ابزار اصلي مطابقت دارد.

Title: تأثير تمرينات ثبات دهنده مركزي بر درد و عملكرد بیماران زن مبتلا به کمردرد مزمن غیر اختصاصی

http://jrrs.mui.ac.ir/index.php/jrrs/article/view/394

مقدمه: کمردرد یک مشکل بزرگ اجتماعی با هزینه‌های هنگفت و مشکل بهداشت در کشورهای صنعتی است. بیش از 20 نوع درمان برای کمردرد مزمن وجود دارد. دست‌یابی به یک برنامه درمانی و توان‌بخشی جهت بهبود سریع‌تر بیماران مبتلا به کمردرد مزمن همواره مورد توجه بوده است. لذا هدف از پژوهش حاضر، ارزیابی تأثير يك دوره تمرينات ثبات دهنده مركزي بر درد و عملكرد بیماران زن مبتلا به کمردرد مزمن بود. مواد و روش‌ها: تعداد 30 بیمار زن غير ورزشكار مبتلا به كمردرد مزمن، با علت ناشناخته (سن 2/5 ± 3/36 سال، وزن 3/8 ± 6/68 كيلوگرم، قد 6/4 ± 8/160سانتي‌متر) که به بیمارستان نفت اهواز جهت مداوا مراجعه کرده بودند به عنوان نمونه‌های تحقیق در این مطالعه شرکت نمودند. بیماران به صورت تصادفي و تساوی در دو گروه تجربي و شاهد تقسيم شدند. در ابتدا ميزان درد و عملکرد بیماران از طريق پرسش‌نامه‌هاي VAS (Visual analogue scale) و Oswestry اندازه‌گيري شد. سپس بیماران گروه تجربی به مدت 12 جلسه به صورت یک روز در میان به تمرينات ثبات دهنده مركزي پرداختند. اما بیماران گروه شاهد به هیچ گونه مدالیته نپرداختند. بعد از این مدت دوباره درد و عملکرد بیماران هر دو گروه مورد ارزیابی قرار گرفت. به منظور آناليز داده‌ها از 17SPSS استفاده شد و سطح معنی‌داري نيز 05/0 > P در نظر گرفته شد. یافته‌ها: نتایج تحقیق بهبود معنی‌داری (05/0 > P، 13 = t) در میزان درد بیماران گروه تجربی (33/0 ± 5/6 در مقابل 45/0 ± 1/2) به دنبال تمرینات ثبات دهنده نشان داد. اما در بیماران گروه شاهد (46/0 ± 27/5 در مقابل 48/0 ± 3/5) تفاوت معنی‌داری مشاهده نشد (05/0 < P). در خصوص عملکرد تفاوت معنی‌داری (05/0 > P، 8/3 = t) بین قبل و بعد از تمرینات در بیماران گروه تجربی (3/3 ± 3/26 در مقابل 3/4 ± 4/14) مشاهده شد و به طور قابل ملاحظه‌ای در عملکردشان بهبود حاصل شد، اما در بیماران گروه شاهد (7/4 ± 1/33 در مقابل 2/4 ± 12/33) تفاوت معنی‌داری مشاهده نشد (05/0 < P). نتیجه گیری: از یافته‌های این تحقيق می‌توان نتیجه‌گیری کرد که تمرينات ثبات دهنده مركزي در کاهش درد و بهبود عملكرد در بیماران مبتلا به کمردرد مزمن تأثير شایانی داشته است. لذا پيشنهاد مي‌شود که در برنامه درمانی بیماران مبتلا به کمردرد مزمن تمرينات ثبات دهنده مرکزی نیز در نظر گرفته شود.

Title: بررسی مقدماتی نگرش، دانش و عملکرد آسیب‌شناسان گفتار و زبان نسبت به فعالیت مبتنی بر شواهد

http://jrrs.mui.ac.ir/index.php/jrrs/article/view/321

مقدمه: فعالیت مبتنی بر شواهد (Evidence based practice یا EBP) به ترکیب تحقیقات با مهارت‌های بالینی گفته می‌شود که در این فرایند به نظرات مراجعه‌کنندگان هم توجه می‌شود. در سال‌های اخیر از آسیب‌شناسان گفتار و زبان انتظار می‌رود که بتوانند تصمیم‌های بالینی خود را با توجه به شواهد معتبر توجیه کنند. از این رو هدف این مطالعه بررسی نگرش، دانش و عملکرد آسیب‌شناسان گفتار و زبان از EBP و رابطه آن با متغیرهای شخصی و درمانی است. مواد و روش‌ها: این مطالعه مقطعي، توصيفي- تحليلي و گذشته‌نگر است. در این تحقیق 40 نفر از آسيب‌شناسان گفتار و زبان در دسترس شهر اصفهان به وسیله پرسش‌نامه‌ "بررسي ميزان نگرش و عملكرد نسبت به فعاليت مبتني بر شواهد" مورد ارزیابی قرار گرفتند. اطلاعات به دست آمده با استفاده از آزمون‌هاي آمار توصيفي كه شامل محاسبه درصد فراواني پاسخ‌هاي مختلف است، مورد بررسي قرار گرفت. برای آنالير داده‌ها از نرم‌افزار 16SPSS استفاده شد. یافته‌ها: 79 درصد از فارغ‌التحصیلان و دانشجویان مقاطع کارشناسی و کارشناسی ارشد نگرش مثبتی نسبت به EBP داشتند. 90 درصد شرکت‌کنندگان در این مطالعه پاسخ دادند که هیچ گونه آموزشی در این زمینه نداشتند. 32 درصد از آسیب‌شناسان گفتار و زبان بیان کردند که به طور معمول کمتر از 2 مقاله در طی یک ماه ‌خواندند و 3/1 از پاسخ‌دهندگان نیز گزارش کردند که کمتر از 2 بار در ماه از منابع برای تصمیم‌گیری بالینی استفاده نمودند. تنها نیمی از نمونه‌ها چه در خانه و چه در محل کار، به اطلاعات آنلاین دسترسی داشتند. نتیجه گیری: این یافته‌ها، نیاز به آموزش بیشتر EBP و ارایه راهکارهای مناسب جهت افزایش کاربرد آن نشان داد.

Title: بررسي اثربخشی آموزش دستورالعمل‌های مدرسه کمر در بهبود درد و سطح ناتواني کارکنان مبتلا به كمردرد مزمن

http://jrrs.mui.ac.ir/index.php/jrrs/article/view/169

مقدمه: ابتلا به كمردرد به لحاظ بي‌تحركي طولاني مدت و وضعیت‌های ناصحيح بدني در ميان کارکنان دفتري پشت ميز نشين اختلالي شايع است كه به واسطه آن افراد از عوارض فيزيكي، رواني، اجتماعي و حرفه‌اي رنج مي‌برند. در اين مطالعه جهت بررسی اثربخشی آگاهی از وضعیت‌های مناسب بدنی حین فعالیت‌های روزمره در تخفيف درد و سطح ناتواني کارکنان مبتلا به كمردرد، از يك پمفلت آموزشي تصويري استفاده شد. مواد و روش‌ها: براي اجراي يك مداخله سه ماهه، مطالعه‌اي نيمه تجربي به صورت قبل و بعد طراحي گرديد و در آن 40 كارمند دفتري50-25 ساله با ميانگين سني 7/35 سال مبتلا به كمردرد مزمن از ميان 6 اداره دولتي به روش نمونه‌گيري آسان انتخاب شدند. در ابتداي مطالعه، درد و سطح ناتواني آن‌ها با مقياس VAS (Visual analog scale) و پرسش‌نامه ناتواني اسوستری (Oswestry disability questionnaire) اندازه‌گيري شد. سپس دستورالعمل‌های پمفلت در محل کار فرد طی یک جلسه 20 دقیقه‌ای توسط كارشناس فیزیوتراپیست آموزش داده شد. در پایان ماه اول و دوم پس از آموزش، اصول آموزش داده شده به فرد یادآوری و انجام آن کنترل گردید. در انتهای ماه سوم دوباره متغیرهای درد و سطح ناتوانی ارزیابی شد. تحلیل داده‌ها با آزمون Paired t-test صورت گرفت. یافته‌ها: در رابطه با شدت درد، تفاوت میانگین نمرات VAS قبل و بعد از آموزش معنی‌دار شد (0001/0 = P). همچنین تفاوت میانگین نمرات سطح ناتوانی قبل و بعد از آموزش نیز معنی‌دار شد (0001/0 = P). نتیجه گیری: رعايت دستورالعمل‌هاي به كار گرفته شده در پمفلت طي مدت 3 ماه مي‌تواند با تخفیف درد و کاهش سطح ناتوانی در کارکنان همراه باشد.

Title: بررسی تأثیر 8 هفته تمرینات هوازی بر الگوی توزیع فشار کف پایی بیماران مبتلا به دیابت نوروپاتی

http://jrrs.mui.ac.ir/index.php/jrrs/article/view/269

مقدمه: نوروپاتی حسی– حرکتی با تحت تأثیر قرار دادن اعصاب حسی و حرکتی منجر به کاهش حس عمقی، درک حرکت و موقعیت مفصل می‌شود. نتیجه آن تغییر الگوی راه رفتن از حالت طبیعی و تغییر مسیر مرکز فشار و اعمال فشار غیر طبیعی در نواحی متاتارسال می‌شود که در طی زمان احتمال بروز زخم پا را به همراه دارد. مطالعه حاضر به منظور بررسی تأثیر تمرینات هوازی بر فشار کف پا در حین راه رفتن در بیماران نوروپاتی مبتلا به دیابت انجام شد. مواد و روش‌ها: جامعه آماری شامل بیماران مبتلا به دیابت، مبتلا به عارضه نوروپاتی بودند که 30 نفر از آن‌ها به عنوان نمونه آماری با روش نمونه‌گیری در دسترس انتخاب و به طور داوطلبانه در دو گروه مساوی تمرین و شاهد قرار گرفتند که در پایان به 12 نفر در هر گروه کاهش یافتند (گروه تمرین 07/9 ± 85/55 سال و Kg/m2 09/10 ± 50/29 = Body mass index یا BMI، گروه شاهد 53/5 ± 66/54 سال و  Kg/m2 26/4 ± 55/31 = BMI). میزان حداکثر فشار کف پا در حین راه رفتن آزمودنی‌ها قبل و بعد از برنامه تمرینی با دستگاه Foot scan (RSscan) در ده ناحیه آناتومیکی اندازه‌گیری شد تا نواحی متأثر از تمرین به طور دقیق مشخص شود. گروه تمرین به مدت 8 هفته، هفته‌ای 3 جلسه و 1 ساعت در هر جلسه به انجام ورزش هوازی (بدون کنترل شدت تمرین) پرداختند. گروه شاهد در این مدت به زندگی معمول خود ادامه دادند. یافته‌ها: داده‌های فشار در پیش و پس آزمون با روش ANOVA با اندازه‌گیری‌های مکرر در نواحی ده‌گانه بررسی شد و نتایج نشان‌گر کاهش معنی‌دار فشار در نواحی متاتارسال و میان پا در گروه تمرین بود (05/0 ≤ P). در حالی که در گروه شاهد هیچ تغییر معنی‌داری مشاهده نشد. نتیجه گیری: این مطالعه نشان داد که بیماران مبتلا به دیابت با انجام فعالیت‌های ورزشی می‌توانند صدمات ناشی از نوروپاتی مانند زخم پا را که به دلیل توزیع غیر طبیعی فشار به خصوص در ناحیه متاتارسال به وجود می‌آید، کاهش دهند.

Title: بررسی تأثیر مدیریت جامع کیفیت (FOCUS PDCA) بر عملکرد بخش کاردرمانی دانشکده توان‌بخشی شیراز

http://jrrs.mui.ac.ir/index.php/jrrs/article/view/320

مقدمه: توان‌بخشی کودکان فلج مغزی از مباحث مهم در حیطه کاردرمانی می‌باشد. برنامه توان‌بخشی این کودکان فرایندی مستمر و درازمدت است که والدین را با مشکلاتی نظیر ماندن در لیست انتظار، جابجایی و حمل و نقل کودک و مسایل مالی مواجه می‌کند. این مشکلات می‌تواند پی‌گیری و اعتماد خانواده را به برنامه‌های درمانی کاهش دهد و دستیابی به هدف را به تعویق بیندازد. در پژوهش حاضر جهت ارتقای سیستم و رفع مشکلات ذکر شده، یکی از ابزارهای مدیریت جامع کیفیت به نام FOCUS-PDCA (Find, Organization, Clarify, Understanding, Select, Plan, Do, Check, Act) اجرا شد و تأثیر آن بر عملکرد بخش کاردرمانی دانشکده علوم توان‌بخشی دانشگاه شیراز مورد بررسی قرار گرفت. مواد و روش‌ها: مطالعه حاضر مداخله‌ای شبه تجربی بود که در دو گروه آزمون و شاهد انجام گرفت. در هر گروه 15 کودک فلج مغزی و والدین آن‌ها 12 جلسه تحت بررسی قرار گرفتند. گروه شاهد از خدمات کاردرمانی سنتی در کلینیک استفاده نمودند. پس از تشکیل تیم ارتقاء در گروه آزمون به جزء خدمات کلینیکی، آموزش والدین و انجام تمرینات در منزل نیز مورد استفاده قرار گرفت. رضایت‌مندی والدین در انتها توسط پرسش‌نامه محقق ساخته و عملکرد حرکتی درشت کودکان قبل و بعد از مداخله توسط مقیاس پی‌بادی (Peabody scale) در دو گروه مورد ارزیابی قرار گرفت. یافته‌ها: تحلیل داده‌ها حاکی از تأثیر معنی‌دار روشFOCUS-PDCA  در بخش کاردرمانی بر عملکرد حرکتی کودکان فلج مغزی (05/0 > P) و رضایت‌مندی والدین (001/0 > P) بود. نتیجه گیری: پژوهش حاضر نشان داد که اجرای روشFOCUS-PDCA  منجر به ارتقای عملکرد حرکتی درشت کودکان فلج مغزی و افزایش رضایت‌مندی والدین مراجعه کننده به کاردرمانی می‌گردد. پیشنهاد می‌شود که این روش در دیگر حیطه‌های درمانی و توان‌بخشی و از نظر تأثیر آن بر شاخص‌های دیگر نیز مورد ارزیابی قرار گیرد.

Title: مقایسه استئوآرتریت زانو در ورزشکاران نخبه بازنشسته فوتبال و افراد غیر ورزشکار

http://jrrs.mui.ac.ir/index.php/jrrs/article/view/203

مقدمه: امروزه فوايد انجام فعالیت‌های بدني در پيش‌گيري از بیماری‌های مزمني هم چون استئوآرتريت پيشنهاد می‌شود، اما خطرهایی که فعالیت بدنی شدید ممکن است بر روی افراد و به خصوص سیستم اسکلتی- عضلانی آنان داشته باشد، همچنان ناشناخته است. هدف از این مطالعه، مقایسه درجه استئوآرتريت رادیوگرافیک و درد زانو، ميزان علایم، مشكلات عملكرد حركتي در فعالیت‌های روزانه، ورزشي، تفريحي و کیفیت زندگي در ورزشکاران نخبه بازنشسته فوتبال و افراد غیر ورزشکار بوده است. مواد و روش‌ها: در این مطالعه 15 ورزشکار مرد حرفه‌ای بازنشسته فوتبال و 15 فرد غير ورزشکار به عنوان آزمودني به صورت هدفمند انتخاب شدند. تشخیص بیماری با تأیید علایم بالینی و رادیولوژیکی (Kellgren-Lawrence) توسط پزشک ارتوپد انجام و از پرسش‌نامه جهاني و بومي‌سازي شده پیامد صدمات زانو و استئوآرتریت (Knee injury osteoarthritis outcome score و یا KOOS) استفاده شد. براي تجزيه و تحليل داده‌ها از آزمون Independent t در سطح معنی‌داري 05/0 ≤ P استفاده گردید. یافته‌ها: بالاتر بودن میانگین درجه استئوآرتریت رادیوگرافیک زانو (001/0P = ) و پایین‌تر بودن میانگین شدت درد زانو (002/0P = )، ميزان علایم (001/0 > P)، مشكلات عملكرد حركتي در فعالیت‌های روزانه (001/0P = )، ورزشي، تفريحي (001/0 > P) و کيفيت زندگي (001/0 > P) در گروه ورزشکار نسبت به غیر ورزشکار (كه نشان دهنده وخیم‌تر بودن وضعیت در گروه ورزشکار مي‌باشد)، اختلاف معنی‌داری را نشان داد. نتیجه گیری: ورزش فوتبال به صورت حرفه‌ای و در سطح قهرمانی احتمال ابتلا به بیماری استئوآرتریت زانو را در بین ورزشکاران نخبه بازنشسته افزایش می‌دهد.

Title: اثربخشی آموزش گروهی مهارت‌های زندگی جمعی بر ارتقای مهارت‌هاي روزمره بیماران اسکیزوفرنیا

http://jrrs.mui.ac.ir/index.php/jrrs/article/view/261

مقدمه: هدف از پژوهش حاضر بررسي اثربخشی آموزش گروهی مهارت‌های زندگی جمعی بر ارتقای مهارت‌هاي روزمره بیماران مبتلا به اسکیزوفرنیا مرکز آموزشی- درمانی روان‌پزشکی رازی تهران بود. مواد و روش‌ها: برای بررسي اثربخشی مداخله مذكور از طرح آزمایشی پیش ‌آزمون- پس ‌آزمون همراه با گروه شاهد استفاده شد. 24 نفر از بیماران اسکیزوفرنیا به طور تصادفی به دو گروه آزمایش (12 = n) و گروه شاهد (12 = n) تقسیم شدند. برای گردآوری داده‌ها از چک‌لیست مهارت‌های زندگی روزانه كه توسط کربلایی اسماعیل در سال 1376 تهيه شده بود و همچنين از پرسش‌نامه مشخصات دموگرافیک استفاده گردید. جهت تجزیه و تحلیل داده‌ها از ANCOVA تک متغیره استفاده شد. سطح معنی‌داري 05/0 > P در نظر گرفته شد. در ضمن، داده‌هاي حاصل از پژوهش با بسته نرم‌افزاري 16SPSS تجزيه و تحليل شد. یافته‌ها: آموزش گروهی مهارت‌های زندگی جمعی باعث ارتقای مهارت‌های زندگي روزمره بیماران اسکیزوفرنیا در گروه آزمایش پس از آموزش برنامه شد (05/0 > P). به این معنی که گروه آزمایش مهارت‌های زندگي بهتری را در مقایسه با گروه شاهد نشان دادند. نتیجه گیری: یافته‌های منتج از این پژوهش نشان‌گر آن است که مي‌توان از برنامه آموزش گروهی مهارت‌های زندگی جمعی در کنار دارو درمانی برای ارتقای مهارت‌های زندگي و ارتباطی در بیماران اسکیزوفرنیا بهره جست.

Title: افزایش انعطاف‌پذیری عضله همسترینگ: امواج ماورای صوت یا تسهیل حس عمقی؟

http://jrrs.mui.ac.ir/index.php/jrrs/article/view/298

مقدمه: کوتاهی عضله همسترینگ می‌تواند باعث اختلالات وضعیتی و الگوی راه رفتن غیر طبیعی گردد. در سال‌های اخیر، مداخلات درمانی متفاوتی جهت برطرف نمودن کوتاهی عضله همسترینگ مطرح شده‌اند. به همین دلیل، این مطالعه با هدف مقایسه دو نوع روش درمانی امواج ماوراء صوت و تکنیک کشش تسهیل حس عمقی (Hold– Relax) انجام گرفت. مواد و روش‌ها: این مطالعه از نوع نیمه تجربی بود. تعداد 36 آزمودنی به صورت در دسترس انتخاب شدند و به صورت تصادفی به سه گروه شاهد، درمان با امواج ماورای صوت و درمان با تکنیک کششیHold– Relax  تقسیم شدند. دو گروه آخر برنامه‌های درمانی مربوط به خود را به مدت 8 هفته دنبال نمودند و جهت اندازه‌گیری دامنه حرکتی مفاصل هیپ و زانو از گونیامتر استاندارد استفاده گردید. جهت تحلیل داده‌های حاصل از این تحقیق از تحلیل واریانس یک سویه در بسته نرم‌افزاری 19SPSS و با سطح معنی‌داری (05/0 > P) استفاده شد. یافته‌ها: یافته‌های این مطالعه نشان داد که هر دو روش درمانی به طور معنی‌داری باعث افزایش دامنه حرکتی فلکشن هیپ و اکستنشن زانو می‌شوند (001/0 > P). با این حال گروه تحت درمان با تکنیک (Hold– Relax یا HR) در مقایسه با گروه تحت درمان با ماوراء صوت (Ultrasound یا US) به طور معنی‌داری با افزایش بیشتری در دامنه حرکتی فلکشن هیپ و اکستنشن زانو رو به رو شدند (001/0 > P). نتیجه گیری: به منظور افزایش انعطاف پذیری عضله همسترینگ، تکنیک تسهیلی HR اثربخشی بیشتری دارد. با این حال مطالعات بیشتری جهت بررسی اثر درمان ترکیبی US و تکنیک HR و سایر تکنیک‌های تسهیلی حس عمقی نیاز است.

Title: بررسی میزان استفاده از «کار» در مداخلات کاردرمان‌گران اصفهان و تهران در مقایسه با کاردرمان‌گران آمریکا

http://jrrs.mui.ac.ir/index.php/jrrs/article/view/396

مقدمه: کار «Occupation» شامل تکالیف روزمره و فعالیت‌های هدفمندی است که هر فرد در آن‌ها مشارکت می‌کند و برای وی معنی و یا ارزش شخصی یا ذهنی خاصی دارد. مفهوم «کار» هسته مرکزی کاردرمانی را تشکیل می‌دهد. کاردرمان‌گران به وسیله تسهیل مشارکت در کار‌ فرصتی برای تکامل فرد ایجاد می‌کنند و استقلال وی را افزایش می‌دهند. با توجه به اهمیت استفاده از کار در کاردرمانی و با توجه به این که تاکنون در ایران پژوهشی در زمینه کار صورت نگرفته است، این سؤال مطرح می‌شود که آیا کاردرمان‌گران در ایران نیز به استفاده از کار در مداخلات خود التزام دارند؟ در این پژوهش به بررسی میزان استفاده از کار توسط کاردرمان‌گران ایران در مقایسه با کاردرمان‌گران آمریکا پرداخته شده است. مواد و روش‌ها: این پژوهش به روش کمی پیمایشی انجام شد. ابزار مورد استفاده مقیاس کارِ درمانی (Inpatient rehabilitation–scale of therapeutic occupation) بود که در سال 1998 توسط Sass و Nelson طراحی شده است. این ابزار پس از اعمال تغییراتی مورد استفاده قرار گرفت. جامعه مورد بررسی کاردرمان‌گران از شهر تهران و اصفهان بودند که نام آن‌ها در انجمن کاردرمانی ایران یا سازمان نظام پزشکی ثبت شده بود. در این پژوهش از نمونه‌گیری در دسترس استفاده شد. برای تجزیه و تحلیل داده‌ها، ابزار 17SPSS مورد استفاده قرار گرفت. سطح معنی‌داری در این پژوهش 01/0 در نظر گرفته شد. یافته‌ها: شرکت کنندگان در این پژوهش شامل 70 نفر (31 مرد و 39 زن) با میانگین سنی حدود 29 سال بودند. نتایج این پژوهش با پژوهش صورت گرفته توسط Sass و Nelson در آمریکا در سال 1998 مقایسه شد. میزان t محاسبه شده نشان داد که کاردرمان‌گران آمریکا بیش از کاردرمان‌گران ایران از کار در مداخلاتشان استفاده می‌کردند (001/0 > P). نتیجه گیری: با توجه به نتایج این پژوهش، ملاحظه می‌شود که تفاوت زیادی میان میزان استفاده کاردرمان‌گران از کار در درمان در ایران و آمریکا وجود دارد؛ به طوری که کاردرمان‌گران در آمریکا بیش از کاردرمان‌گران در ایران از کار در درمان استفاده می‌کنند. از دلایلی که می‌توان در تبیین این تفاوت نام برد تفاوت در سابقه حرفه کاردرمانی، شرایط کار بالینی و برنامه‌های آموزشی در ایران و آمریکا است. به نظر می‌رسد جهت پیشرفت بیشتر حرفه کاردرمانی در ایران، آموزش بیشتر و بالینی‌تر در زمینه کار و ایجاد شرایط کاری مناسبتر امری ضروری باشد.

Title: بررسی نگرش دانشجویان نسبت به افراد دارای لکنت

http://jrrs.mui.ac.ir/index.php/jrrs/article/view/242

مقدمه: تحقيقات متعددی در مورد نگرش نسبت به افراد لکنتی انجام شده است. هدف از اين مطالعه بررسی نگرش دانشجويان نسبت به افراد لکنتی بود. مواد و روش‌ها: پژوهش حاضر يک مطالعه توصيفي- تحليلي بود که در بين دانشجويان دانشگاه علوم پزشکي گرگان انجام شد. 104 شرکت کننده يک پرسش‌نامه حاوی 20 ويژگي شخصيتي را در يک مقياس 7-1 درجه‌ای تکميل نمودند. یافته‌ها: در کليه ويژگي‌هاي شخصيتي به جز ويژگي‌های "پرخاش‌گر" (584/0 = P) و"آرام" (151/0 = P) از لحاظ آماری اختلاف معنی‌داري بین افراد لکنتی و افراد غیر لکنتی وجود داشت (05/0 > P). همچنين ميانگين نمرات ويژگي‌های منفی در افراد لکنتی بيشتر بود. نتیجه گیری: اين مطالعه نشان داد که دانشجويان نگرش منفي‌تري نسبت به افراد لکنتي در مقايسه با افراد غير لکنتي دارند.

Title: پیامدهای بازسازی و توان‌بخشی رباط صلیبی قدامی از طریق دو روش گرافت تاندون پاتلار و گرافت تاندون همسترینگ در ورزش‌کاران

http://jrrs.mui.ac.ir/index.php/jrrs/article/view/379

مقدمه: پارگی رباط صلیبی قدامی (Anterior cruciate ligament یا ACL) یکی از شایع‌ترین آسیب‌های زانو می‌باشد که در بین ورزش‌کاران حرفه‌ای اتفاق می‌افتد. انتخاب بهترین گرافت برای ورزش‌کاران دارای ACL ناکارامد اهمیت زیادی دارد. در طی سال‌های اخیر گرافت‌های تاندون پاتلار و همسترينگ برای بازسازی ACL بیشتر مورد استفاده بوده‌اند. اين مطالعه با هدف، بررسي تأثير نوع گرافت بر ميزان عملكرد، تعادل و بازگشت به مسابقات ورزشي از طريق دو روش گرافت تاندون پاتلار (Bony patellar tendon bony graft یا BPTBG) و گرافت تاندون همسترینگ (Hamstring tendon graft یا HTG) انجام گرفت. مواد و روش‌ها: در این پ‍ژوهش نیمه تجربی، 38 ورزش‌کار دارای پارگی ACL به تدریج مورد مطالعه قرار گرفتند. 19 ورزش‌کار با گرافت تاندون پاتلار (BPTBG) و 19 ورزشکار با گرافت تاندون همسترینگ (HTG) 4 لایه، تحت عمل بازسازی قرار گرفتند. برای هر دو گروه یک برنامه بازتوانی تسریعی طراحی و اجرا شد. در پایان ماه‌های سوم، ششم و نهم، متغیرهایی مثل درد قدامی زانو، دامنه حرکتی (ROM)، مقیاس لی‌شلم، تست لی تک پا، آزمون گردش ستاره، یک تکرار بیشینه (1RM)، ناپايداري زانو و میزان بازگشت به مسابقات ارزیابی شد. از آزمون Repeated measure ANOVA و آزمون t برای تجزیه و تحلیل نتایج در سطح 05/0 بهره گرفته شد. یافته‌ها: در پایان ماه‌های 6 و 9 درد قدامی زانو در گروه BPTBG به طور معنی‌داری بیشتر از گروه HTG بود. زانو زدن برای گروه BPTBG در پایان 9 ماه همراه با درد شدیدتر و تورم بیش‌تری بود (05/0 > P). اکستنشن در گروه BPTBG بیشتر از گروه HTG و در فلکشن برعکس بود. ناپايداري قدامی زانو در گروه HTG بیشتر از گروه BPTBG بود، ولی اختلاف معنی‌دار به ثبت نرسيد (05/0 > P). در لی تک پایی و تعادل، گروه BPTBG دارای عملکرد ضعیف‌تری بودند. نمره مقیاس لی‌شلم در گروه HTG به طور معنی‌داری بهتر از گروه BPTBG بود و گروه HTG به سطح بالاتری از رقابت بازگشت (05/0 > P). نتیجه گیری: نتایج تحقیق نشان داد که استفاده از گرافت همسترینگ به دلیل داشتن پیامدهای جانبی کمتر و بازگشت مطمئن‌تر و سریع‌تر به سطح اولیه ورزشی، گرافت مناسبی برای بازسازی ACL می‌باشد.

Title: ارتباط بین ناهنجاری کف پای صاف با زاویه Q و زانو درد

http://jrrs.mui.ac.ir/index.php/jrrs/article/view/219

مقدمه: طبق نظریه زنجیره حرکتی جاندا (Janda’s kinetic chain theory) اختلال به وجود آمده در یک مفصل می‌تواند مفاصل نزدیکتر را نیز متأثر سازد. هدف پژوهش حاضر، بررسی ارتباط بین ناهنجاری کف پای صاف با زاویه Q و زانو درد در کشتی‌گیران سبک آزاد ایران بود. مواد و روش‌ها: برای اجرای این تحقیق 20 کشتی‌گیر تیم ملی با میانگین سنی 86/0 ± 11/19 سال، وزن 4/18 ± 5/70 کیلوگرم، قد 1/9 ± 2/173 سانتی‌متر انتخاب شدند. برای اندازه‌گیری کف پای صاف از افت ناوی (افت استخوان ناويكولار- روش Brody و همکاران)، برای اندازه‌گیری زاویه Q زانو از گونیامتر و برای اندازه‌گیری درد زانو از خط‌کش درد (VAS) Visual analog scale استفاده گردید. برای تجزیه و تحلیل آماری از برنامه آماری 18SPSS و آزمون آماری Pearson در سطح معنی‌دار 05/0.استفاده شد. همچنین از آزمون  Interclass correlation coefficient (ICC) برای بررسی تکرارپذیری داده‌های اندازه‌گیری شده استفاده شد. یافته‌ها: با توجه به نتایج تحقیق رابطه معنی‌داری بین ناهنجاری کف پای صاف و زانو درد در بین کشتی‌گیران یافت شد (686/0 = r). رابطه معنی‌دار بین افزایش زاویه Q در پای برتر و زانو درد مشاهده شد (949/0 = r). همچنین رابطه معنی‌دار ضعیفی بین افزایش زاویه Q در پای برتر و کف پای صاف مشاهده شد (278/0 = r). نتیجه گیری: با توجه به نتایج حاصل از این تحقیق می‌توان نتیجه‌گیری کرد که بر اساس سیستم زنجیره حرکتی، ناهنجاری کف پای صاف شاید باعث چرخش جانبی پاتلا و در نهایت افزایش زاویه Q شده و این عامل منجر به درد زانو در کشتی‌گیران سبک آزاد می‌شود.

Title: تأثير تمرينات پيلاتس بر روي برخي عوامل فيزيولوژيکي و آمادگي جسماني زنان غير ورزشکار داراي اضافه وزن

http://jrrs.mui.ac.ir/index.php/jrrs/article/view/485

مقدمه: پيلاتس يکي از روش‌هاي تمريني جديد است که به نظر مي‌رسد در کاهش وزن و در نتيجه کاهش بيماري‌هاي قلبي- عروقي و همچنين کاهش پرفشاري خون مؤثر باشد. بنابراين هدف از تحقيق حاضر، تعيين اثر تمرينات پيلاتس بر برخي عوامل فيزيولوژيکي و آمادگي جسماني زنان غير ورزشکار داراي اضافه وزن بود. مواد و روش‌ها: آزمودني‌هاي اين تحقيق نيمه تجربي، ٣٠ نفر از دانشجويان دختر غير ورزشکار داراي اضافه وزن با ميانگين شاخص توده بدني (٨۴/١ ± ٢۵/٢۶ کيلوگرم بر متر مربع) بودند که به طور داوطلبانه در اين تحقيق شرکت کردند و به طور تصادفي به دو گروه تجربي و شاهد تقسيم شدند. آزمودني‌هاي گروه تجربي به مدت ٨ هفته، ٣ جلسه در هفته و هر جلسه يک ساعت در تمرينات پيلاتس شرکت کردند. گروه شاهد تمرين خاصي را انجام ندادند و به همان روند قبل از تحقيق خود ادامه دادند. قبل و بعد از دوره تمريني ويژگي‌هايي شامل: وزن، شاخص توده بدني، درصد چربي (ضخامت چربي زير پوستي ۴ ناحيه: سه سر بازو، شکم، فوق خاصره، ران)، توده چربي، توده بدون چربي، محيط کمر، توان هوازي، فشارخون سيستول و دياستول در زمان استراحت، قدرت دست برتر، استقامت عضلاني شکم و انعطاف‌پذيري (بشين و برس و خيزتنه) اندازه‌گيري شد. به منظور تجزيه و تحليل داده‌ها از نرم‌افزار SPSS و روش‌هاي آماري ANCOVA و آزمون Dependent t-test استفاده شد. یافته‌ها: درصد چربي، توده چربي بدن و محيط کمر در گروه تجربي پس از دوره تمريني به طور معني‌داري کاهش يافته بود (٠۵/٠ > P). همچنين توان هوازي، استقامت عضلات شکم، انعطاف‌پذيري (بشين و برس و خيزتنه) و قدرت دست برتر گروه تجربي به طور معني‌داري بيشتر از گروه شاهد بود (٠۵/٠ > P). وزن کل بدن، شاخص توده بدني، توده بدون چربي و فشارخون سيستول و دياستول گروه تجربي تفاوت معني‌داري را نسبت به گروه شاهد نشان نداد (٠۵/٠ < P). نتیجه گیری: با توجه به نتايج به دست آمده، به نظر مي‌رسد که انجام تمرينات پيلاتس به مدت ٨ هفته، ٣ جلسه در هفته و هر جلسه يک ساعت، مي‌تواند باعث کاهش درصد چربي، توده چربي و محيط کمر و بهبود ويژگي‌هايي نظير انعطاف‌پذيري، قدرت دست، استقامت عضلاني ناحيه شکم و توان هوازي شود.

Title: تأثير تمرينات ورزشي ترکيبي بر کيفوز پشتي و لوردوز کمري نوجوانان کم‌توان ذهني

http://jrrs.mui.ac.ir/index.php/jrrs/article/view/388

مقدمه: تحرک افراد کم‌توان ذهنی در مقايسه با افراد سالم كمتر است و اين مسأله شاید آن‌ها را در معرض خطر ناتوانايي‌هاي جسماني و حركتي قرار ‌دهد. جذب اين افراد به برنامه‌هاي ورزشي مي‌تواند از خطرات فوق بكاهد. در اين مطالعه، تأثير 8 هفته تمرينات ورزشي ترکيبي‌ بر کيفوز پشتي و لوردوز کمري نوجوانان کم‌توان ذهني بررسي شد. مواد و روش‌ها: مطالعه از نوع نيمه تجربي بود. جامعه آماری پژوهش شامل30 نوجوان کم‌توان ذهني (سن 21/1 ± 9/16 سال، قد 0/9 ± 172 سانتي‌متر، وزن 76/16 ± 4/63 کيلوگرم و بهره هوشي 07/5 ± 86/62) بود كه مشكل افزايش زاويه‌هاي لوردوز و کيفوز داشتند. آن‌ها بر اساس نمره هوش به طور تصادفي جفت شدند و به دو گروه 15 نفري شاهد و تجربي تقسيم گردیدند. گروه شاهد به روال عادي زندگي خود ادامه داد، ولي گروه تجربي در يك برنامه تمريني شامل 8 هفته (3 جلسه 60 دقيقه‌اي در هفته) تمرينات ترکيبي آمادگي جسماني و حرکات اصلاحي شركت كرد. با استفاده از خط‌کش منعطف، زاويه‌هاي كيفوز و لوردوز پيش و پس از مداخله پژوهشي اندازه‌گيري شد. تجزيه و تحليل آماري داده‌ها با استفاده از نرم‌افزار 17SPSS و توسط آزمون ANOVA براي اندازه‌هاي تکرار شده انجام شد. یافته‌ها: زاويه لوردوز در گروه‌هاي شاهد و تجربي به ترتيب0/1+ و 4/3- درجه تغيير داشت. اين به معناي وجود تعامل معنی‌دار آماري بين دو گروه می‌باشد [001/0 > P، 6/35 = (28، 1)F]. زاويه كيفوز نيز وضعيتي مشابه داشت و نمرات پس‌آزمون گروه‌هاي شاهد و تجربي به ترتيب 2/0+ و 6/2- درجه تغيير نشان دادند [014/0 = P، 6/9 = (28، 1)F]. نتیجه گیری: در مطالعه حاضر، نوجوانان کم‌توان ذهني جذب يك برنامه تمرين ورزشي تركيبي شامل تمرينات آمادگي جسماني و حرکات اصلاحي شدند. نتايج به دست آمده حاكي از اثربخشي تمرينات به كار رفته در جهت كاهش معنی‌دار زاويه‌هاي کيفوز پشتي و لوردوز کمري بود. به نظر مي‌رسد استفاده از برنامه‌هاي فعاليت بدني و تمرينات ورزشي‌ مشابه براي افراد کم‌توان ذهني قابل توصيه باشد.
